# HYPK-ing up COP1 to put the brakes on photomorphogenesis

**DOI:** 10.1093/plcell/koag322

**Published:** 2026-10-22

**Authors:** Jiajun Wang, Yueyao Wang

**Affiliations:** Assistant Features Editor, The Plant Cell, American Society of Plant Biologists; School of Life Sciences, Xiamen Key Laboratory of Plant Genetics, Xiamen University, Xiamen 361102, China; School of Advanced Agricultural Sciences, Peking University, Beijing 100871, China

Seedlings undergo photomorphogenesis after germination in the light, typically characterized by short hypocotyls, expanded cotyledons, and functional chloroplast development. Photomorphogenesis is essential for seedling establishment and survival following emergence from the soil. CONSTITUTIVELY PHOTOMORPHOGENIC1 (COP1), a RING-finger E3 ubiquitin ligase, acts as a central repressor of photomorphogenesis, and its loss of function results in a constitutive photomorphogenic phenotype in darkness (Deng et al. 1992). In vivo, COP1 associates with SUPPRESSOR OF PHYA (SPA) proteins to form the CUL4-DDB1^COP1/SPA^ E3 ubiquitin ligase complex, which promotes the ubiquitination and degradation of positive regulators of photomorphogenesis, including the transcription factor ELONGATED HYPOCOTYL5 (HY5) (Lau and Deng 2012). The activity of the COP1/SPA complex is precisely and tightly controlled by multiple regulatory mechanisms involving photoreceptors and COP1 SUPPRESSOR (CSU) (Zhou and Deng 2025); however, whether additional factors regulate COP1/SPA complex activity remains largely unknown.

Huntingtin yeast partner K (HYPK) is an evolutionarily conserved regulator of the ribosome-associated N^α^-terminal acetyltransferase A (NatA) complex, which catalyzes the N-terminal acetylation of a large fraction of cellular proteins. In Arabidopsis, HYPK promotes NatA activity and contributes to the stability and proteostasis of its substrate proteins (Miklankova et al. 2022; Gong et al. 2024). In a recent study, Yuan Gao and colleagues (Gao et al. 2026) identified HYPK as a positive regulator of COP1 activity that represses photomorphogenesis (see Fig. 1).

**Figure 1:**
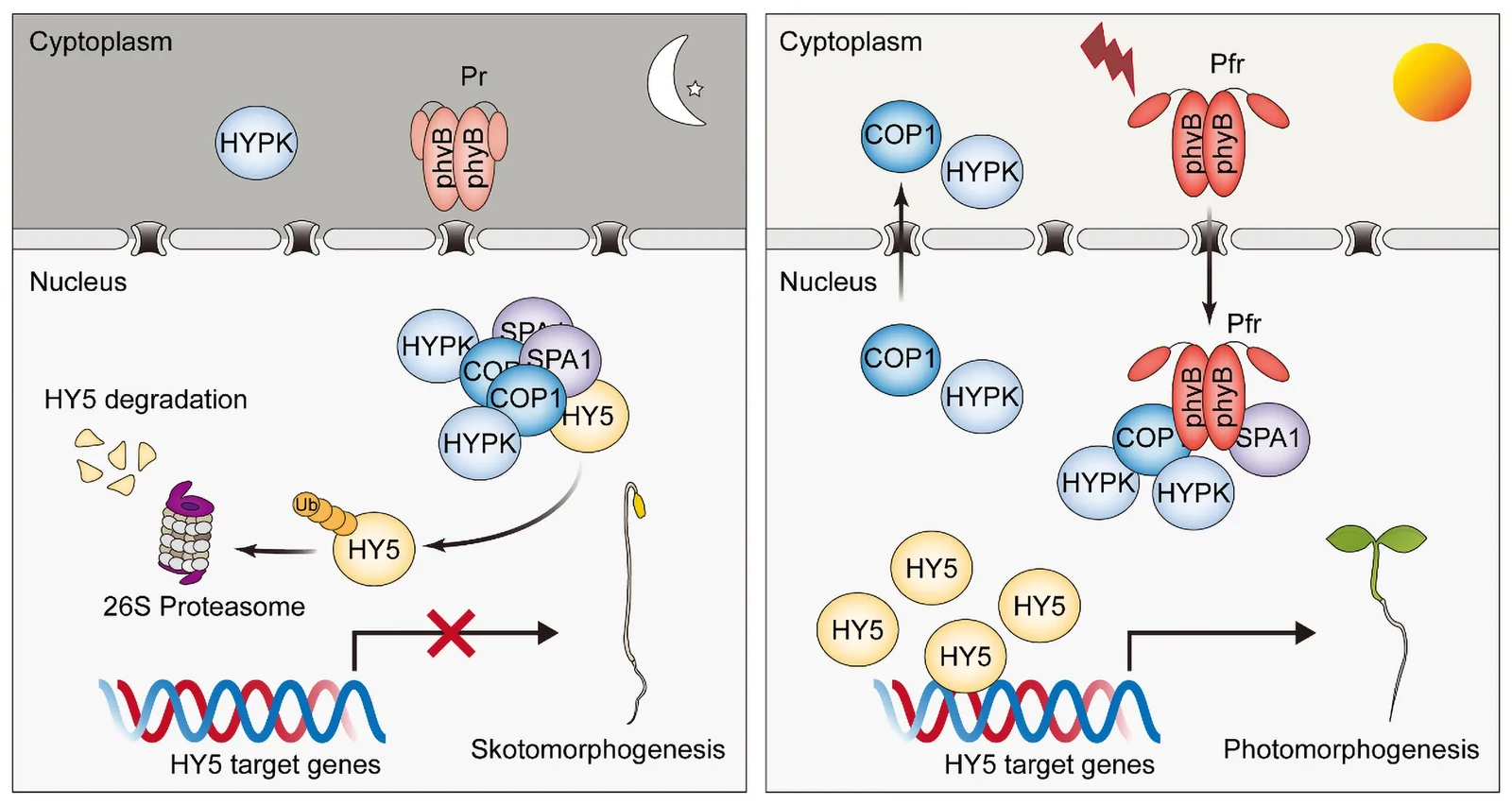
Working model illustrating how HYPK promotes COP1 activity to repress photomorphogenesis. In darkness, HYPK promotes the interaction between COP1 and SPA proteins and enhances COP1/SPA complex-mediated ubiquitination and degradation of HY5, thereby repressing the expression of photomorphogenesis-associated genes and maintaining skotomorphogenesis. Upon exposure to light, the capacity of HYPK to promote HY5 degradation is attenuated, while nuclear accumulation of phyB suppresses COP1-mediated HY5 degradation, allowing photomorphogenesis to proceed. Reprinted from Gao et al. (2026), Figure 7.

The authors initially identified HYPK as a candidate phytochrome B (phyB)-interacting protein through immunoprecipitation–mass spectrometry. To investigate whether HYPK participates in light signaling transduction, the authors generated 2 HYPK genome-edited lines using CRISPR/Cas9, including a 3-amino-acid deletion mutant (HYPKΔN) and a null allele. Phenotypic analyses revealed that both lines exhibited shortened hypocotyls and enhanced photomorphogenesis in darkness and under continuous red, far-red, and blue light. Genetic analyses further indicated that HYPK acts downstream of phyB and CRY1 to regulate light-induced inhibition of hypocotyl elongation. Consistent with its role downstream of phyB and CRY1, HYPK itself is subject to light regulation: its transcript levels were repressed by light in *phyA*, *phyB*, or *cry1* mutants, respectively, whereas its protein abundance was modestly increased upon light exposure.

Because the *hypk* phenotype resembles that of *cop1* mutants, the authors next examined whether HYPK interacts with COP1. Pull-down, firefly luciferase complementation imaging, and co-immunoprecipitation assays demonstrated that HYPK directly interacts with COP1. Notably, the interaction between the 3-amino-acid deletion mutant HYPKΔN and COP1 was substantially reduced. To further explore how HYPK represses photomorphogenesis, the authors investigated whether HYPK regulates COP1-mediated HY5 ubiquitination and degradation. Loss of HYPK function markedly reduced HY5 ubiquitination and increased HY5 protein stability. Further analyses showed that HYPK did not affect the interaction between COP1 and HY5 but enhanced COP1-mediated HY5 ubiquitination, while the loss-of-function mutant HYPKΔN failed to promote COP1-mediated HY5 ubiquitination. These results indicate that HYPK promotes HY5 degradation through its interaction with COP1, thereby maintaining skotomorphogenesis in darkness.

Genetic analyses further established HY5 as a downstream target of HYPK, as loss of *HY5* suppressed the enhanced photomorphogenic phenotypes of hypk mutants. Moreover, the enhanced phenotypes of *hypk cop1* double mutants were largely suppressed by loss of *HY5*, indicating that HYPK and COP1 cooperatively regulate HY5 to repress photomorphogenesis.

Interestingly, although HYPK did not affect COP1 protein abundance in darkness, it promoted COP1 nuclear accumulation during the dark-to-light transition. In hypk mutants, fewer COP1 photobodies formed, with lower fluorescence intensity but larger diameters than in wild-type seedlings, both in darkness and during the early stages of the dark-to-light transition. In contrast, HYPK did not affect phyB protein abundance but suppressed its nuclear accumulation during the dark-to-light transition. In the *hypk* mutant, phyB photobodies were larger and exhibited higher fluorescence intensity, whereas *HYPK* overexpression reduced both parameters. Consistent with these observations, *hypk* further enhanced, whereas HYPK overexpression attenuated, the short-hypocotyl phenotype of phyB-overexpressing seedlings.

In addition to regulating the nuclear localization and photobody properties of both COP1 and phyB, HYPK reduced the interaction between COP1 and phyB while promoting the interaction between COP1 and SPA1. Thus, HYPK appears to promote COP1 activity through multiple mechanisms to enhance the repression of photomorphogenesis.

Together, these findings identify HYPK as a previously uncharacterized repressor of photomorphogenesis. In darkness, HYPK promotes COP1–SPA association, maintains COP1 photobody organization, and enhances COP1-mediated ubiquitination and degradation of HY5, thereby repressing the expression of photomorphogenesis-associated genes and maintaining skotomorphogenesis (see Fig. 1). During the dark-to-light transition, HYPK protein levels increase, leading to the suppression of phyB nuclear accumulation, photobody formation, and the phyB–COP1 interaction, attenuating photomorphogenesis. This study expands the function of HYPK beyond its established role as a regulator of the NatA complex and co-translational N-terminal acetylation.

## Recent related articles in *The Plant Cell*

- Jiang et al. (2025) showed that photoexcited CRY1 directly interacts with ATG8 to inhibit ATG8-mediated autophagic degradation of HY5, thereby promoting photomorphogenesis in Arabidopsis.
- Yuan et al. (2026) revealed that BEE1/2/3, whose light-induced degradation is mediated by the 26S proteasome, interact with phyA/phyB and SPAs to fine-tune the phyA/B–COP1/SPA1 signaling hub by stabilizing PIF3 and disrupting the phyA/B–SPA1 association, thereby negatively regulating photomorphogenesis under excessive light.
- In the commentary by Eckardt et al. (2024), coauthors Ning Wei and Giovanna Serino address the question of how plants use ubiquitin-mediated proteolysis to regulate photomorphogenesis.
- Willige et al. (2024) reviewed recent advances on phytochrome B (phyB) photobodies, highlighting that these light-regulated nuclear domains form via liquid–liquid phase separation through multivalent interactions and function as biochemical hotspots that regulate gene expression at multiple levels in light and temperature signaling.

## Data Availability

N/A.
